# The Liver Protection Effects of Maltol, a Flavoring Agent, on Carbon Tetrachloride-Induced Acute Liver Injury in Mice via Inhibiting Apoptosis and Inflammatory Response

**DOI:** 10.3390/molecules23092120

**Published:** 2018-08-23

**Authors:** Wei Liu, Zi Wang, Jin-gang Hou, Yan-dan Zhou, Yu-fang He, Shuang Jiang, Ying-ping Wang, Shen Ren, Wei Li

**Affiliations:** 1 College of Chinese Medicinal Materials, Jilin Agricultural University, Changchun 130118, China; liuwei3996@126.com (W.L.); wangzi8020@126.com (Z.W.); houjg2014@kaist.ac.kr (J.-g.H.); zyd0331@126.com (Y.-d.Z.); jiangshuang0503@hotmail.com (S.J.); yingpingw@126.com (Y.-p.W.); 2 Intelligent Synthetic Biology Center, Daejeon 34141, Korea; 3 College of Management, Changchun University of Chinese Medicine, Changchun 130117, China; hyf_1992@163.com; 4 National & Local Joint Engineering Research Center for Ginseng Breeding and Development, Changchun 130118, China

**Keywords:** maltol, carbon tetrachloride, liver injury, inflammation, apoptosis, oxidative stress

## Abstract

The purpose of this research was to evaluate whether maltol could protect from hepatic injury induced by carbon tetrachloride (CCl_4_) in vivo by inhibition of apoptosis and inflammatory responses. In this work, maltol was administered at a level of 100 mg/kg for 15 days prior to exposure to a single injection of CCl_4_ (0.25%, i.p.). The results clearly indicated that the intrapulmonary injection of CCl_4_ resulted in a sharp increase in serum aspartate transaminase (AST) and alanine transaminase (ALT) activities, tumor necrosis factor-α (TNF-α), irreducible nitric oxide synthase (iNOS), nuclear factor-kappa B (NF-κB) and interleukin-1β (IL-1β) levels. Histopathological examination demonstrated severe hepatocyte necrosis and the destruction of architecture in liver lesions. Immunohistochemical staining and western blot analysis suggested an accumulation of iNOS, NF-κB, IL-1β and TNF-α expression. Maltol, when administered to mice for 15 days, can significantly improve these deleterious changes. In addition, TUNEL and Hoechst 33258 staining showed that a liver cell nucleus of a model group diffused uniform fluorescence following CCl_4_ injection. Maltol pretreatment groups did not show significant cell nuclear condensation and fragmentation, indicating that maltol inhibited CCl_4_-induced cell apoptosis. By evaluating the liver catalase (CAT), glutathione (GSH), superoxide dismutase (SOD) activity, and further using a single agent to evaluate the oxidative stress in CCl_4_-induced hepatotoxicity by immunofluorescence staining, maltol dramatically attenuated the reduction levels of hepatic CAT, GSH and SOD, and the over-expression levels of CYP2E1 and HO-1. In the mouse model of CCl_4_-induced liver injury, we have demonstrated that the inflammatory responses were inhibited, the serum levels of ALT and AST were reduced, cell apoptosis was suppressed, and liver injury caused by CCl_4_ was alleviated by maltol, demonstrating that maltol may be an efficient hepatoprotective agent.

## 1. Introduction

The liver is a crucial organ in the metabolic system. It can remove many harmful substances and drugs. However, it will be harmed by these harmful substances [1]. Acute liver injury (ALI) caused by toxic chemicals, drugs, or pathogen infections is one of the most life-threatening diseases. Although many drugs have the potential to treat ALI, these are limited by adverse reactions during long-term use [2,3].

The carbon tetrachloride (CCl_4_)—induced hepatotoxicity model is one of the most extensively used in vivo models. It is now recognized that CCl_4_ is a chemical hepatotoxin that can cause oxygen free radical-induced liver damage [4,5]. Oxidative damage and an inflammatory response are caused by CCl_4_ metabolism, which in turn causes severe liver damage and hepatocyte damage associated with necrosis and apoptosis [6]. More importantly, oxidative stress is related to inflammation [7]. It has been well documented that CCl_4_ can induce the occurrence of acute liver injury, the activation of macrophages, and the release of many pro-inflammatory cytokines, including tumor necrosis factor-α (TNF-α), irreducible nitric oxide synthase (iNOS), nuclear factor-kappa B (NF-κB) and interleukin-1β (IL-1β) [8]. Notably, the above inflammatory factors and oxidative stress responses continue to participate in the pathological process of acute hepatic injury, which is a vicious cycle of aggravating liver disease [9,10].

The compound 3-hydroxy-2-methyl-4-pyrone, with the molecular formula C_6_H_6_O_3_, also known as maltol, is one of the maillard reaction products. Maltol is widely disseminated in nature and consumed as a safe and reliable flavor potentiator and food preservative all over the world [11]. In recent years, it was found that maltol exerts excellent protective activity against oxidative stress [12]. Maltol protects against glycation-derived free radicals and exhibits a potential therapeutic application in neuropathies [13,14]. In addition, previous studies demonstrated that maltol can effectively maintain the normal physiological functions of cells by exerting protective effects against the oxidative damage [15] caused by reactive oxygen species (ROS) [16], and is able to prevent kidney damage in diabetic nephropathy [17]. Maltol with iron supplements and other complexes is now frequently used for the treatment of certain diseases [18,19]. Studies have implied that maltol-derived ergonomic compositions may be safe for patients suffering from various tumors [20,21]. Based on these observations, the accumulation of ethanol-induced free radicals and inflammation were ameliorated by antioxidants including maltol, suggesting that maltol may have therapeutic potential for liver injury [22,23,24]. Additionally, little was known about the effect of maltol in CCl_4_-induced acute liver injury in mice. In the present study, we provided experimental evidence of the use of maltol for the treatment of acute liver injury caused by CCl_4_ via inhibiting apoptosis and inflammatory responses.

## 2. Results

### 2.1. Effect of Maltol on Body Weight and Organ Index in Mice

As described in previous studies, the changes of body weights before and after the experiment in mice and the organ coefficients of the liver and kidney were evaluated [25,26]. As shown in the Table 1, CCl_4_-treated mice gained less weight than the normal control. Liver and kidney coefficients were significantly increased following CCl_4_ administration (*p* < 0.05). However, the growth of liver weight and kidneys were significantly inhibited in the maltol administration group.

### 2.2. Effect of Maltol on Serum Aspartate Transaminase (Alt) and Alanine Transaminase (Ast) Activities

**Figure 1:**
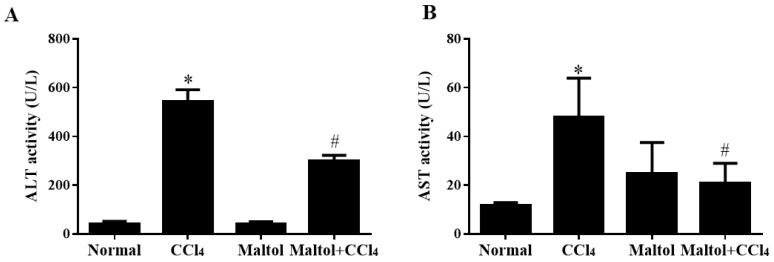
Pretreatment with maltol protected against CCl_4_-induced liver injury: The effect of maltol on serum levels of aspartate transaminase (ALT) (**A**) and alanine transaminase (AST) (**B**), which were provided by Nanjing Jiancheng Bioengineering Research Institute (Nanjing, China). Values are expressed as the mean ± S.D., *n* = 8. * *p* < 0.05 vs. normal group; ^#^ *p* < 0.05 vs. CCl_4_ group.

Maltol could significantly decrease serum aspartate transaminase (ALT) and alanine transaminase (AST) activities, which are two biological indicators for detecting liver function and are the basis for judging liver damage. The effects of maltol on serum AST and ALT activities are shown in Figure 1. In the normal group, ALT and AST activities in serum were 19.48 ± 10.52 and 9.60 ± 1.65 U/L, separately. We found there were no demonstrated changes in AST and ALT activities in the group pretreated with maltol alone compared with the normal group, while the levels of serum ALT and AST were reversed after treatment with maltol for 15 days (*p* < 0.05). Collectively, ALT and AST activity induced by CCl_4_ can be significantly reduced during maltol treatment (both *p* < 0.05).

### 2.3. Maltol Ameliorated CCl_4_-Induced Hepatic Histopathological Changes in Mice

**Figure 2:**
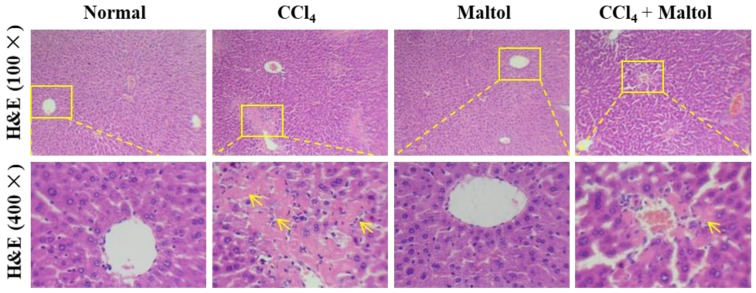
Histological examination of morphological changes in liver tissues of hematoxylin–eosin (H&E) staining. The pale pink area shows the necrotic area. Original magnification: 100× and 400×.

The following figure shows representative photomicrographs of livers collected from mice in normal, experimental and maltol + CCl_4_ groups. We found obvious histological abnormalities induced by CCl_4_ (Figure 2). Obviously, massive hepatocyte necrosis and nuclear shrinkage, as well as the loss of hepatocyte structure around the blood vessels, were significantly alleviated by maltol compared with the model group. As shown in Table 2, hepatic cell necrosis was more clearly noted in the model group compared with the normal group indicated by Ridit analyses (*U* = 3.884, *p* = 0.0001). Nevertheless, the degree of hepatic cell necrosis was apparently improved in the maltol group (*U* = 2.043, *p* = 0.041) compared with the normal group, with no apparent changes in the maltol group (*U* = 0.338, *p* = 0.735).

### 2.4. Maltol Inhibited Cell Apoptosis in CCl_4_-Induced Acute Liver Injury in Mice

**Figure 3:**
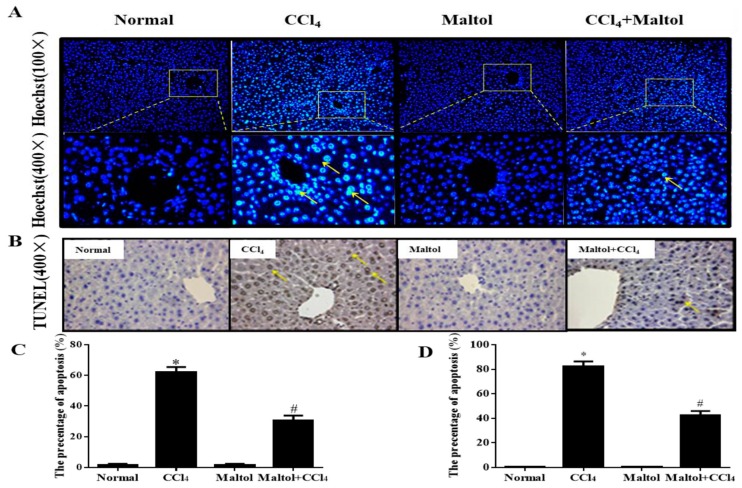
Effects of maltol on CCl_4_-induced inhibited cell apoptosis: Hoechst 33258 (**A**). Original magnification: 100× and 400×. The yellow arrows show apoptosis cells. The mean optical density of Hoechst 33258 staining is shown in (**C**). The effects of maltol on hepatic apoptosis examined with TUNEL is shown in (**B**). Original magnification: 400×. The percentage of positive cells of liver cells stained with TUNEL is shown in (**D**). Values are expressed as the mean ± S.D., *n* = 8. * *p* < 0.05 vs. normal group; ^#^ *p* < 0.05 vs. CCl_4_ group.

In order to determine whether maltol pretreatment inhibited cell apoptosis in CCl_4_-induced ALI in vivo, a Hoechst 33258 staining assay were performed. The results showed that CCl_4_ induced the diffusion of uniform fluorescence, while no significant cell nuclear condensation and fragmentation was detected after maltol pretreatment (Figure 3A). Additionally, the mean optical density of liver cells is shown in Figure 3C.

Next, liver sections were stained using TUNEL colorimetric assay (Figure 3B). The model group showed clearly apoptosis clearly as indicated by arrow heads, which was profoundly ameliorated by maltol. The quantification of cell apoptosis is shown in Figure 3C,D respectively. The data were analyzed by an Image-Pro plus system.

### 2.5. Effects of Maltol on Oxidative Stress Markers

**Figure 4:**
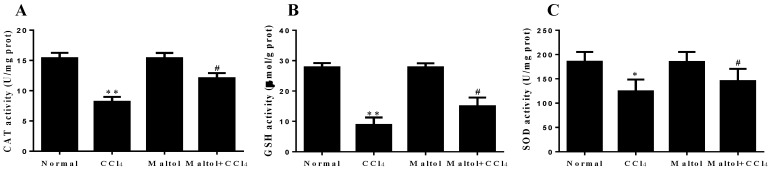
Pretreatment with maltol protected against CCl_4_-induced liver injury: Effects of maltol on the hepatic of catalase (CAT) (**A**), glutathione (GSH) (**B**), superoxide dismutase (SOD) (**C**) in CCl_4_-induced mice; values are expressed as the mean ± S.D., *n* = 8. * *p* < 0.05, ** *p* < 0.01 vs. normal group; ^#^ *p* < 0.05 vs. CCl_4_ group.

**Figure 5:**
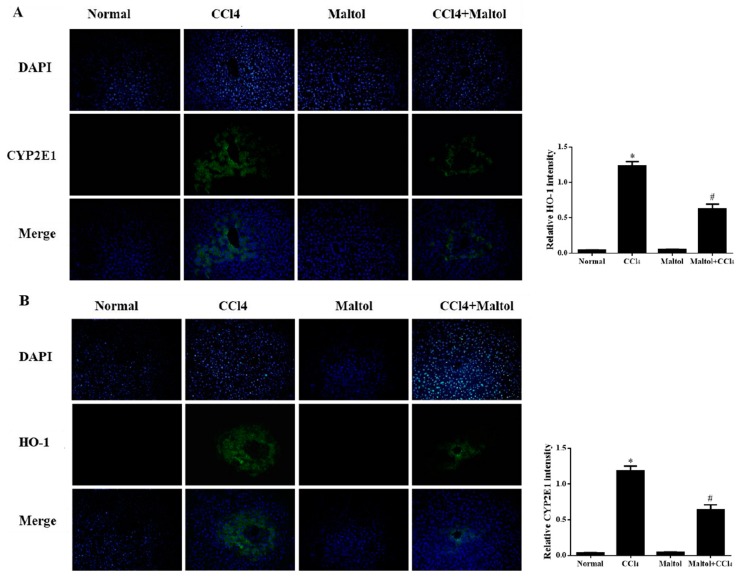
Pretreatment with maltol protected against CCl_4_-induced liver injury: Liver cells stained with immunofluorescence probes of cytochrome P450 E1 (CYP2E1) (**A**) and heme oxygenase-1 (HO-1) (**B**). Representative quantification of immunofluorescence images at 200×. 4,6-Diamidino-2-phenylindole (DAPI) was used as a nuclear counterstain. Values are expressed as the mean ± S.D., *n* = 8. * *p* < 0.05 vs. normal group; ^#^ *p* < 0.05 vs. CCl_4_ group.

As we know, oxidative stress injury is one of the most important mechanisms in the study of chemical-induced hepatotoxicity in mice [27]. The functions of liver endogenous antioxidant enzymes catalase (CAT), glutathione (GSH), superoxide dismutase (SOD) by the induction of CCl_4_ to mice are summarize in the study (Figure 4). Meanwhile, in order to further validate the relationship between oxidative stress and hepatotoxicity in vivo, in the present study, the expression levels of CYP2E1 and HO-1 in liver tissues were analyzed by fluorescence staining. The results showed that positive expression areas of these two markers exerted strong green fluorescence, which demonstrated the oxidative stress injury caused by CCl_4_ exposure in mice. Compared with the normal group, CCl_4_ injection significantly enhanced the overexpression of CYP2E1 and HO-1 in the liver tissues, while following maltol pretreatment ameliorated this elevation, illustrating that maltol exerted anti-oxidative stress injury (Figure 5).

### 2.6. Effect of Maltol on CCl_4_-Induced Inflammatory Response

**Figure 6:**
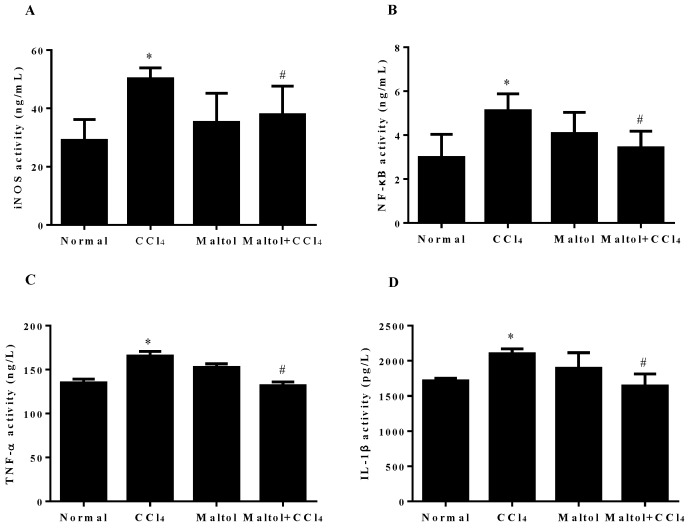
Pretreatment with maltol protected against CCl_4_-induced liver injury: Effects of maltol on the serum levels of irreducible nitric oxide synthase (iNOS) (**A**), nuclear factor-kappa B (NF-κB) (**B**), tumor necrosis factor-α (TNF-α) (**C**) and interleukin-1β (IL-1β) (**D**) in CCl_4_-induced mice; values are expressed as the mean ± S.D., *n* = 8. * *p* < 0.05 vs. normal group; ^#^ *p* < 0.05 vs. CCl_4_ group.

As mentioned above, inflammation response is also associated with CCl_4_-induced liver injury. To analyze the effects of maltol on CCl_4_-induced inflammatory response, the secretion contents of iNOS, NF-κB, TNF-α and IL-1β in serum were measured by enzyme-linked immunosorbent assay (ELISA). As shown in Figure 6, there are extensively up-regulation of hepatic iNOS, NF-κB, TNF-α, and IL-1β after single injection of CCl_4_, especially in IL-1β secretion. Interestingly, these elevation levels of inflammatory mediators were significantly attenuated (*p* < 0.05) by maltol pretreatment. In addition, the maltol alone group did not display significant changes.

**Figure 7:**
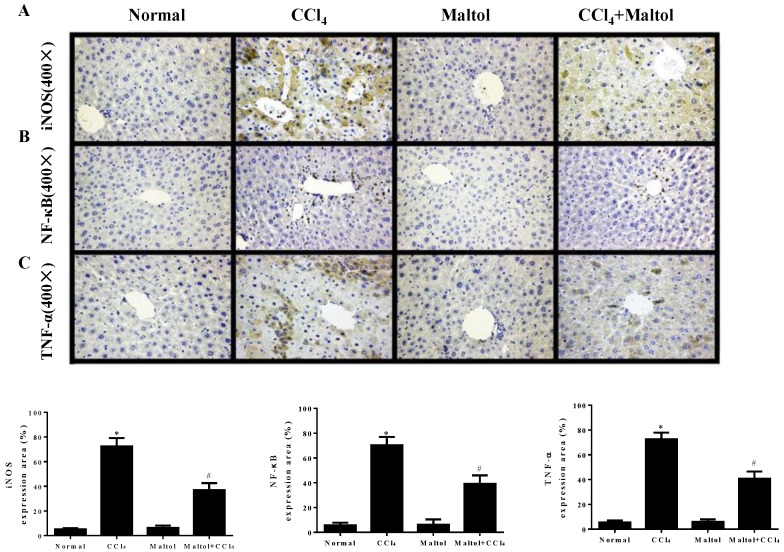
Effects of maltol on the CCl_4_-induced expression of inflammatory mediators: photomicrographs of (**A**): iNOS (1:100), (**B**): NF-κB (1:100), (**C**): TNF-α (1:100) at 400×. From histopathological staining analysis, in normal livers, the serum levels of cytokines expression were negligible, with the CCl_4_ group having a lot of positive staining. The livers of mice receiving CCl_4_ + maltol were similar to controls. The brown spots showed positive. iNOS, inducible nitric oxide synthase; NF-κB, nuclear factor-kappa B; TNF-α, Tumor necrosis factor-α; IL-1β, interleukin-1β. Values are expressed as the mean ± S.D., *n* = 8. * *p* < 0.05 vs. normal group; ^#^ *p* < 0.05 vs. CCl_4_ group.

**Figure 8:**
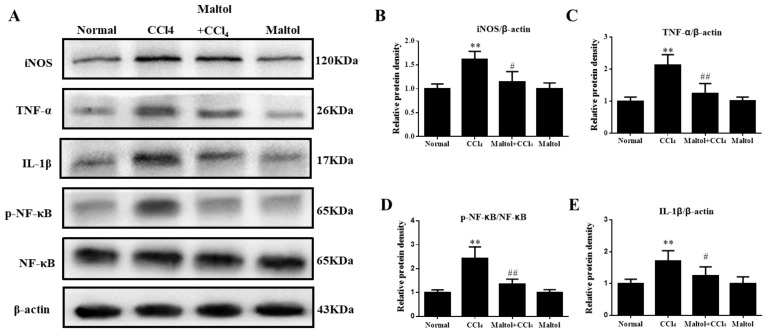
Effects of maltol on CCl_4_-induced expression of inflammatory mediators: western blotting was used to determine the expressions of IL-1β, TNF-α, iNOS and p-NF-κB/NF-κB (p65) to further illustrate the underlying mechanism of maltol protecting the liver from damage. We also used specific primary antibodies, and β-actin protein levels as a loading control (**A**). Quantification of relative protein expression was performed by densitometric analysis (**B**–**E**). Values are expressed as mean ± SD (*n* = 3 in each group). * *p* < 0.05, ** *p* < 0.01 vs. normal group; ^#^ *p* < 0.05, ^##^ *p* < 0.01 vs. CCl_4_-induced group.

In order to confirm the effect of maltol on inflammatory response, we further determined the expression levels of CCl_4_-induced inflammatory mediators by immunohistochemistry and western blotting analysis. As shown in Figure 7, the liver tissues of CCl_4_-treated mice showed positive staining of iNOS, NF-κB and TNF-α. Importantly, pretreatment with maltol for 15 days significantly inhibited the overexpression of TNF-α and iNOS, and suppressed the activation of NF-κB (*p* < 0.01). Importantly, the results from western blot analysis provide the evidence that pretreatment with maltol significantly decreased the protein levels of iNOS, NF-κB, TNF-α and IL-1β (Figure 8).

## 3. Discussion

Studies have shown that CCl_4_ can cause severe kidney damage [28], and our results also suggest that CCl_4_ significantly increases the renal index while significantly increases the liver index, and maltol has an obvious mitigation effect. Our research focuses on acute liver injury induced by CCl_4_. Our study systematically illustrated the protective effects of maltol against hepatotoxicity induced by CCl_4_, which is widely used for screening of the hepatoprotective function of plant extracts and drugs in many rodent models [29]. CCl_4_ could induce severe liver damage accompanied by hepatocellular necrosis and apoptosis. The purpose of the present study was to show determine maltol as a protective candidate against CCl_4_-induced liver injury in mice by inhibiting apoptosis and inflammatory response.

The cause of liver damage induced by CCl_4_ may be linked to the level of transaminase located in the cytoplasm, and a decrease in the liver’s structural integrity can increase the serum level of these enzymes. The toxicity of CCl_4_ is generally dependent on the cleavage of the carbon-chlorine bond to produce trichloromethyl radicals. A trichloromethyl substitute free radical is formed by a free radical reacting rapidly with oxygen, which in turn increases with hepatotoxicity and the subsequent production of liver enzymes, leading to severe liver damage [30]. Additionally, previous studies have demonstrated that serum ALT and AST activities were increased because of CCl_4_ and the membrane integrity of hepatocytes was changed in mice [31,32]. In the present study, maltol significantly inhibited apoptosis and the increase of liver damage indicators, and subsequently alleviated hepatic histological changes in CCl_4_-induced acute hepatic damage in mice. Simultaneously, TUNEL and Hoechst 33258 positive cells, which are evident in the model group, possibly represent the cells undergoing apoptosis induced by CCl_4_. Maltol pretreatment clearly attenuated the apoptosis in the liver-injured mice, suggesting that maltol may potentially serve as a novel option for the treatment of CCl_4_-induced liver injury in mice.

In recent years, research has proven that maltol can effectively relieve the effect of oxidative stress on liver injury in mice [33]. The current research suggests that antioxidant enzymes such as CAT, GSH and SOD, which are scavengers of free radicals in the liver, are the first line of defense against endogenous oxidative injury, more importantly, the levels of CYP2E1 and HO-1 expression are commonly for testing oxidative stress, CCl_4_ is mainly metabolized to highly reactive trichloromethyl free radicals by CYP2E1 and HO-1 [34]. CCl_4_ toxicity immediately induced hepatocytes death, which the subpopulation of CYP2E1 hepatocytes obviously remain in the central vein zone at 12 h after CCl_4_ injection. Liver damage was induced by these reactive free radicals accompanied by the triggering of a chain of cellular events. Previous studies suggested that CYP2E1 suppression could reduce reactive metabolite formation and thus decrease tissue injury [35]. In the present study, maltol significantly suppressed the overexpression of CYP2E1 and HO-1 following CCl_4_ intoxication. The above results clearly indicated that maltol ameliorated CCl_4_-induced liver oxidative stress injury in mice model, which was supported by the findings from alcohol-induced liver injury [33].

More importantly, oxidative stress is often associated with inflammation. CCl_4_ can directly induce oxidative stress and triggers inflammatory cells by exposure to free radicals and toxic debris in mice [30]. Therefore, liver damage might be greatly propagated by releasing various inflammatory mediators from inflammatory cells after oxidative damage activation [36]. TNF-α and IL-1β are the most representative pro-inflammatory cytokines in inflammatory factors, and many studies have also shown that IL-1β and TNF-α play key roles in the development and maintenance of inflammation, while inflammatory cytokines are horizontally increased, which is related to liver disease [36,37]. Inflammatory mediators such as TNF-α are up-regulated and might induce liver damage via multiple cytotoxic mechanisms [38]. TNF-α can induce pleiotropic cytokines produced by a variety of physiological and pathological conditions [39]. This is related to the products of inflammation and fibrosis, and regulates cytokines to a certain extent, but this is not the direct cause of hepatic cell necrosis induced by CCl_4_ [40,41]. Inflammation might directly activate the intracellular death signals and lead to deleterious outcomes [42]. IL-1β is a cytokine that stimulates the development and differentiation of the immune system and promotes inflammation, causing a lasting fever [43]. This study showed that carbon tetrachloride results in considerably increased TNF-α and IL-1β protein levels of in the liver. INOS is an important inflammatory factor, and considered as an inflammatory marker and plays an important role in the development of many inflammatory diseases [44,45]. TNF-α is released from activated Kupffer cells and, to a certain extent, up-regulates iNOS and stimulates the production of nitric oxide (NO), but in excess it causes NO to up-regulate inflammatory responses-mediated CCl_4_-induced acute hepatotoxicity [46]. In this study, iNOS expression was substantially increased in the liver of mice induced by CCl_4_ and may increase NO and produce nitrosative stress, which is a typical response to liver injury. Our results indicated that maltol has a beneficial effect by inhibiting iNOS-mediated acute liver injury caused by CCl_4_, which is consistent with our previous results that maltol exerted the anti-inflammation effects on alcohol-induced liver injury [33].

Signaling pathways can indicate the overexpression of the transcription factors of related factors, such as the induction of the activation of NF-κB and other proinflammatory genes [47,48]. NF-κB is a nuclear transcription factor and could induce the expressions of many genes such as in apoptosis, viral replication, tumorigenesis and various autoimmune diseases. The expression of these large numbers of genes plays an important part in the regulation of health [49,50]. NF-κB up-regulates the protein expressions of TNF-α, IL-6 and iNOS [51]. In the present study, the increased levels of NF-κB induced by CCl_4_ in mice was suppressed by pretreatment with maltol. Previous works clearly indicated that TNF-α is initiated by TNF-α receptor 1 (TNFR1), and its activation caused by CCl4 exposure induces the increased expression of transcriptional factors, activator protein 1 (AP-1) and nuclear factor kappa B (NF-kB). In addition, CCl4 exposure upregulated the expression level of toll-like receptor 4 (TLR4) [52]. Similarly, previous reports also confirmed that MAPKs as a large family of seine/threonine kinases can also largely mediate the inflammatory signaling from the cell surface to nucleus [53,54]. Therefore, in the present work, the analysis of AP-1 expression in TLR4-MAPKs signal pathway will easily understand the protective effect of maltol on CCl_4_ induced liver injury.

**Figure 9:**
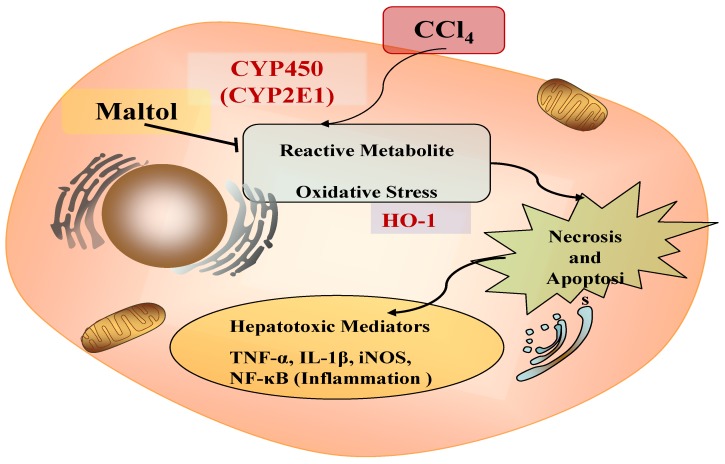
The schematic diagram of mechanism underlying ameliorative effects of maltol against CCl_4_-induced liver injury.

In summary, we concluded that maltol has anti-oxidative stress, anti-inflammatory and anti-apoptosis effect in CCl_4_-induced liver injury. Its possible mechanism of action is shown in Figure 9.

## 4. Materials and Methods

### 4.1. Chemicals and Reagents

The maltol was isolated and purified in our previous research [33], and the purity was 98.0% determined using the HPLC method. The function of the liver was analyzed by commercial assay kits, including AST, ALT, CAT, GSH, SOD and histopathology methods including H&E, which were provided by Nanjing Jiancheng Bioengineering Research Institute (Nanjing, China). A specific two-site sandwich enzyme-linked immunosorbent assay (ELISA) was used to observe the level of iNOS, NF-κB, IL-1β and TNF-α, which were provided by MSK Biological Technology (Wuhan, China) and R&D systems (Minneapolis, MN, USA). The antibodies of rabbit monoclonal anti-mouse iNOS, NF-κB/p-NF-κB (p65), TNF-α, IL-β, CPY2E1, and HO-1 were provided by Cell Signaling Technology (Danvers, MA, USA). All other chemicals used were all analytical grade and from Beijing Chemical Factory.

### 4.2. Animals

Male ICR mice (8 weeks old), weighting 22–25 g, were purchased from YISI Experimental Animal Co., Ltd. with Certificate of Quality No. of SCXK (JI) 2011-0004 (Changchun, China). Animals were maintained under standardized conditions of 50 ± 5% relative humidity, temperature (25 ± 2 °C) in a 12 h light and dark cycle. All animals were fed a standard laboratory diet and ad libitum water.

All experiments were conducted in accordance with the Guide for the Care and Use of Laboratory Animals (Ministry of Science and Technology of China, 2006). All experimental protocols were approved by the Ethical Committee for Laboratory Animals of Jilin Agricultural University (Permit No.: ECLA-JLAU-17098).

### 4.3. Animal Treatment and Experimental Protocol

**Figure 10:**
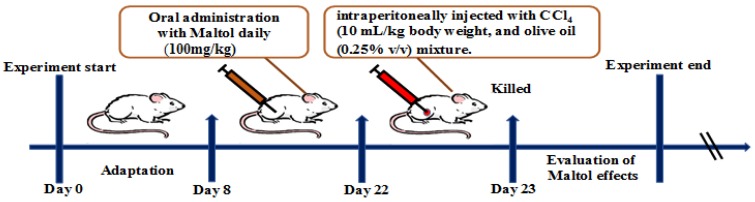
The experiment of pretreatment with maltol protected against CCl_4_-induced liver injury design illustrations.

All experimental animals were randomly divided into four groups (*n* = 8). (1) The normal group and (2) the CCl_4_ group: the normal and CCl_4_ groups ensured respect for received saline once daily for 15 days. One hour after the final saline intervention, mice were injected with CCl_4_ intraperitoneally (10 mL/kg body weight, and olive oil (0.25% *v*/*v*) mixture; (3) The maltol group: mice received maltol (100 mg/kg, disbanded in saline, i.g.); (4) CCl_4_ + maltol group (100 mg/kg body weight) were treated by oral gavage for 15 days. One hour after maltol treatment, mice were treated with CCl_4_ (10 mL/kg body weight), and an olive oil (0.25% *v*/*v*) mixture. Twelve hours after injection, blood samples were gathered and allowed to clot for 45 min at room temperature. Then, the serum was centrifuged (3500 rpm, 10 min, and 4 °C) and stored at −20 °C for biochemical analysis including AST and ALT activities and the secretion levels of type iNOS, NF-κB, TNF-α and IL-1β. All mice were killed. The whole bodies, and segregated livers and spleens were weighed. A respectable piece of liver tissue from the left lobe of the liver was fixed in 10% buffered formalin solution (*m*/*v*) for histopathological analysis and the remaining liver tissues were stored at −80 °C for western blot analysis, as shown in Figure 10.

### 4.4. Determination of Liver Enzymes

The ALT and AST activities in serum, and levels of CAT, GSH and SOD in liver homogenates were measured by commercial kits according to the manufacturer’s instructions (Nanjing Jiancheng Institute of Biotechnology, Nanjing, China). The value of each sample was calculated according to the standard.

### 4.5. Assay for Inflammatory Markers

The ELISA kits for TNF-α and IL-1β were purchased from R&D Systems (Minneapolis, MN, USA) and iNOS and NF-κB were obtained from MSK Biological Technology (Wuhan, china), according to the protocol provided by the manufacturer under prescribed 450 nm conditions in an ELISA reader (Bio-Rad, Hercules, CA, USA).

### 4.6. Histopathological Analysis

Liver tissues were fixed in 10% buffered formalin. They were embedded in paraffin and sectioned for 5 μm thicknesses, then subjected to hematoxylin–eosin (H&E) staining. The histopathological characters were used for the assessment of histological changes of the liver, including by using a Nikon TE2000 fluorescence microscope (Nikon, Japan). Representative images were presented.

### 4.7. Hoechst 33258 Staining

Hoechst 33258 staining was performed by using 5-μm thick tissue sections, and this was performed as described above [53]. Briefly, we randomly selected four sections in each group, and they were rehydrated by xylene and aqueous alcohol solutions carefully, and then were washed by PBS three times before being stained by the dye. Nuclei were visualized under UV excitation and photographed under a fluorescent microscope (Olympus BX-60, Tokyo, Japan). Image-Pro plus 6.0 (Media Cybernetics, Rockville, MD, USA) was used to quantify Hoechst 33258 staining.

### 4.8. TUNEL Assay

In order to detect apoptosis, a TUNEL assay was performed (Roche Applied Science, Shanghai, China) in the liver sections. In the beginning, the sections which were randomly selected were treated with 20 μg/mL of proteinase K in distilled water for 10 min at room temperature. Briefly, paraffin-embedded sections were deparaffinized with the standard protocol. The selected slides were incubated in methanol containing 3% hydrogen peroxide for 20 min and then were incubated with the equilibration buffer and terminal deoxynucleotidyl transferase to block endogenous peroxidase. Finally, sections were incubated with anti-digoxigenin peroxidase conjugate. Peroxidase activity in each tissue section was shown by the application of diaminobenzidine. Sections were countersigned with hematoxylin.

### 4.9. Immunohistochemistry Analysis

Immunohistochemical analysis was performed as previously described [54]. Briefly, the 5-μm thick section samples were embedded in paraffin to be deparaffinized and dehydrated with a series of xylene and aqueous alcohol solutions, respectively. After antigen retrieval in citrate buffer solution (0.01 M, pH 6.0) for 20 min, the slides were washed three times with TBS (0.01 M, pH 7.4) and incubated with 1% bovine serum albumin for 1 h. The blocking serum was tapped off, and the sections were incubated in a humidified chamber at 4 °C overnight with primary antibodies against TNF-α (1:200), iNOS (1:100) and NF-κB (1:100) (Cell Signaling Technology), followed by secondary antibody for 30 min. Substrate was added to the sections for 30 min followed by DAB staining and haematoxylin counter-staining. Positive staining was defined mainly as a brownish-yellow color in the nucleus of the cells. The immunostaining intensity was analyzed by light microscopy (Olympus BX-60, Tokyo, Japan). The immunohistochemical signal was assessed by estimating the area of the objects and the medium pixel intensity per object, as for the optical density (OD).

### 4.10. Immunofluorescence Analysis

Immunofluorescence staining was conducted to assess the expression of CYP2E1 and HO-1 (Cell Signaling Technology, Danvers, MA, USA) in CCl_4_-induced hepatotoxicity directly, the sections were incubated with primary antibodies against rabbit anti-mouse CYP2E1 antibody (1:200) overnight at 4 °C. The next day, after washing with PBS, the slides were exposed to the DyLight 488 labeled secondary antibody (BOSTER, Wuhan, China) for 30 min at room temperature. Then nuclear was counterstained using 4,6 diamidino-2-phenylindole (DAPI). Immunofluorescence staining were visualized using a Leica microscope (Leica TCS SP8, Solms, Germany).

### 4.11. Western Blot Analysis

Western blot analysis was performed as previously described [55]. Protein samples from liver tissues were lysed in 12% SDS-PAGE gel and were lysed with RIPA solution. An electrophoretically transferred buffer was performed to transfer the protein on gel into PVDF membranes, and then the membranes were blocked with 5% non-fat milk for more than 3.5 h and incubated with primary antibodies which were listed as follows: iNOS (1:500), TNF-α (1:500), IL-1β (1:500), NF-κB/p-NF-κB (*p*65) (1:500) and β-actin (1:2000) purchased from Cell Signaling Technology (Danvers, MA, USA) overnight at 4 °C. After washing three times by PBS, the membranes were incubated with the secondary antibodies at room temperature for 1 h. Signals were developed with enhanced chemiluminescence (ECL) substrate (Pierce Chemical Co., Rockford, IL, USA), and analyzed using Quantity One software (Bio-Rad Laboratories, Hercules, CA, USA).

### 4.12. Statistical Analysis

All commercial assays were used in duplicate, and data is expressed as the means ± standard deviation (S.D.). The statistical significance of the differences was analyzed by *t*-test and one-way ANOVA. Followed a Bonferroni post hoc test. *p* values of less than 0.05 or 0.01 were considered to be significant. Statistical significance was produced through GraphPad Prism 6.0.4 (ISI^®^, Philadelphia, PA, USA).

## 5. Conclusions

Altogether, oxidative stress is closely related to inflammation, it is proved that maltol has a significant inhibitory effect on the expression of CYP2E1 and HO-1, which can reduce radicals formation. Meantime, the present study also found that pretreatment with maltol effectively alleviated CCl_4_-induced acute liver injury via the inhibition of apoptosis and inflammatory responses. This demonstrates that maltol has potential as a hepatoprotective agent in cases of chemically induced acute liver injury.

## Figures and Tables

**Table 1:** Effects of maltol on body weight and organ weight in mice.

Groups	Dosage (mg/kg)	Weight (g)		Organ Indices (mg/g × 100)	
		Initial	Final	Liver	Kidney
**Normal**	—	30.7 ± 1.06	30.34 ± 1.02	1.41 ± 0.06	0.45 ± 0.02
**CCl_4_**	—	30.4 ± 1.14	28.78 ± 1.06	1.55 ± 0.20 *	0.50 ± 0.04 *
**Maltol**	100	30.3 ± 1.20	29.96 ± 0.89	1.45 ± 0.05	0.43 ± 0.06 ^#^
**CCl4 + Maltol**	100	30.1 ± 1.13	30.25 ± 1.36	1.43 ± 0.44 ^#^	0.45 ± 0.03 ^#^

Note: values are expressed as the mean ± standard deviation (S.D.), *n* = 8; * *p* < 0.05 vs. Normal group; ^#^ *p* < 0.05 vs. CCl_4_ group.

**Table 2:** Pathological changes in the liver and Ridit analysis.

Groups	Dosage (mg/kg)	*n*	Necrocytosis Grade					Score	Ridit Analysis
			0	1	2	3	4		
**Normal**	—	8	8	0	0	0	0	0	0.28
**CCl_4_**	—	8	0	2	3	3	0	17	0.84 *
**Maltol**	100	8	7	1	0	0	0	1	0.33
**CCl_4_ + Maltol**	100	8	3	4	1	0	0	6	0.55 ^#^

Note: We used H & E staining to evaluate the degree of hepatocyte necrosis, and they were classified according to the following rulers. Ridit analysis was used to analyze the date. Values represent the mean ± S.D., *n* = 8. * *p* < 0.05 vs. normal group; ^#^ *p* < 0.05 vs. CCl_4_ group. Grading standards can be divided into level 0, level 1, level 2, level 3 and level 4, which illustrate no necrocytosis, normal in the liver cells; cells containing necrocytosis of no more than 1/4; cells containing necrocytosis of no more than 1/2; cells containing necrocytosis of no more than 3/4; and almost all cells containing necrocytosis, respectively. Level 0 scored 0 mark, Level 1 scored 1 mark, Level 2 scored 2 marks, Level 3 scored 3 marks, Level 4 scored 4 marks.

## Abbreviations

The following abbreviations are used in this manuscript:

**ALT**	Alanine aminotransferase
**iNOS**	Inducible nitric oxide synthase
**TNF-α**	Tumor necrosis factor-α
**NO**	Nitric oxide
**HO-1**	Heme oxygenase-1
**CAT**	Catalase
**SOD**	Superoxide dismutase
**AST**	Aspartate aminotransferase
**NF-κB**	Nuclear factor-kappa B
**IL-1β**	Interleukin-1β
**CYP2E1**	Cytochrome P450 E1
**CCl_4_**	Carbon tetrachloride
**GSH**	Glutathione
